# Association between body mass index and localized prostate cancer management and disease‐specific quality of life

**DOI:** 10.1002/bco2.197

**Published:** 2022-11-02

**Authors:** Nathan L. Samora, Christopher J. D. Wallis, Li‐Ching Huang, Jacob E. Tallman, Zhiguo Zhao, Karen Hoffman, Alicia Morgans, Matthew Cooperberg, Michael Goodman, Sheldon Greenfield, Ann S. Hamilton, Mia Hashibe, Sherrie Kaplan, Brock O'Neil, Lisa E. Paddock, Antoinette Stroup, Xiao‐Cheng Wu, Tatsuki Koyama, David F. Penson, Daniel A. Barocas

**Affiliations:** ^1^ School of Medicine Vanderbilt University Nashville Tennessee USA; ^2^ Division of Urology University of Toronto, Mount Sinai Hospital Toronto Canada; ^3^ Department of Biostatistics Vanderbilt University Medical Center Nashville Tennessee USA; ^4^ Department of Urology Vanderbilt University Medical Center Nashville Tennessee USA; ^5^ Department of Radiation Oncology The University of Texas MD Anderson Center Houston Texas USA; ^6^ Dana Farber Cancer Institute Harvard Medical School Boston Massachusetts USA; ^7^ Department of Urology University of California San Francisco California USA; ^8^ Department of Epidemiology Emory University Rollins School of Public Health Atlanta Georgia USA; ^9^ Department of Medicine University of California Irvine Irvine California USA; ^10^ Department of Population and Public Health Sciences Keck School of Medicine at the University of Southern California Los Angeles California USA; ^11^ Department of Family and Preventative Medicine University of Utah School of Medicine Salt Lake City Utah USA; ^12^ Department of Urology University of Utah Health Salt Lake City Utah USA; ^13^ Department of Epidemiology Rutgers Cancer Institute of New Jersey New Brunswick New Jersey USA; ^14^ Department of Epidemiology Louisiana State University New Orleans School of Public Health New Orleans Louisiana USA; ^15^ Geriatric Research Education and Clinical Center Veterans Affairs Tennessee Valley Healthcare System Nashville Tennessee USA

**Keywords:** active surveillance, obesity, patient reported outcome measures, prostatectomy, prostatic neoplasms, radiotherapy, watchful waiting

## Abstract

**Purpose:**

The purpose of this work is to describe the association between body mass index (BMI) and (1) management option for localized prostate cancer (PCa) and (2) disease‐specific quality of life (ds‐QoL) after treatment or active surveillance.

**Subjects/patients and methods:**

We analysed data from men with localized PCa managed with radical prostatectomy (RP), radiation therapy (RT), or active surveillance (AS) in a prospective, population‐based cohort study. We evaluated the association between BMI and management option with multivariable multinomial logistic regression analysis. The association between BMI and ds‐QoL was assessed using multivariable longitudinal linear regression. Regression models were adjusted for baseline domain scores, demographics, and clinicopathologic characteristics.

**Results:**

A total of 2378 men were included (medians [quartiles]: age 64 [59–69] years; BMI 27 kg/m^2^; 77% were non‐Hispanic white); 29% were obese (BMI ≥ 30). Accounting for demographic and clinicopathologic features, BMI ≥ 28 kg/m^2^ was inversely associated with the likelihood of receiving RP (compared with RT) and became statistically significant at BMI ≥ 33 kg/m^2^ (maximum adjusted relative risk ratio = 0.80, 95% CI 0.67 to 0.95, *p* = 0.013 for BMI ≥ 33 vs. 25). Conversely, BMI was not significantly associated with the likelihood of receiving AS compared with RT. After stratification by management option, obese men who underwent definitive treatment were not found to have clinically worse ds‐QoL. Obese men initially on AS appeared to have worse urinary incontinence than nonobese men, but this was not significant on an *as‐treated* sensitivity analysis.

**Conclusions:**

Among men with localized PCa, those with BMI ≥ 33 kg/m^2^ were less likely to receive surgery than radiation. Obesity was not associated with ds‐QoL in men undergoing definitive treatment, nor in men who remained on AS.

## 1 INTRODUCTION

Men with localized prostate cancer (PCa) decide among the guideline‐recommended management options of radical prostatectomy (RP), radiation therapy (RT), and active surveillance (AS), based on factors such as disease progression‐risk, age and life expectancy, other comorbidities, treatment side effect profiles, personal preferences, and physician recommendations. For many patients, these modalities confer similar long‐term oncologic survival,^1^ increasing the importance of considering treatment‐related morbidity during shared‐decision making. Post‐treatment urinary, sexual, and bowel function vary based on management option^2^ and anticipated quality of life (QoL) outcomes may influence management option.

Higher body mass index (BMI) is associated with increased intra‐ and peri‐operative complications for patients undergoing RP,^3^, ^4^, ^5^ and physicians may worry that obesity exacerbates respiratory sufficiency in Trendelenburg position.^6^ Still, the association between obesity and disease‐specific QoL (ds‐QoL) after PCa treatment is less certain,^5^, ^7^, ^8^, ^9^ and few studies have assessed to what degree obesity affects treatment choices and counselling. Prior analyses on the effect of obesity on ds‐QoL after localized PCa therapy have had mixed results and have been limited by brief follow‐up and an overemphasis on outcomes after open and laparoscopic RP.^9^, ^10^, ^11^, ^12^ There have been few investigations in men receiving robotic‐assisted radical prostatectomy (RARP) despite this modality being the contemporary standard.^4^, ^5^ Fewer studies have investigated the impact of BMI on ds‐QoL in men who receive RT and AS.^13^

Assumptions about the effect of BMI on post‐treatment ds‐QoL may affect patient counselling and treatment choice. Accordingly, we evaluated the association between BMI, management option, and ds‐QoL outcomes following treatment for localized PCa using data from the prospective, population‐based Comparative Effective Analysis of Surgery and Radiations (CEASAR) study. We hypothesized that obese men are more likely to receive RT than RP and experience worse urinary and sexual function after treatment than nonobese men.

## 2 SUBJECTS/PATIENTS AND METHODS

### 2.1 Study population

The CEASAR study recruited men with clinically localized PCa from five population‐based Surveillance, Epidemiology, and End Results (SEER) Program registries (Atlanta, Georgia; Los Angeles, California; Louisiana; New Jersey; Utah) and the observational Cancer of the Prostate Strategic Urologic Research Endeavor (CaPSURE) prostate cancer registry from 2011 to 2012 as previously described.^14^, ^15^, ^16^ Men were eligible if they were younger than 80 years, had a Prostate Specific Antigen (PSA) level less than 50 ng/ml, had clinical stage T1 or T2 PCa without nodal or metastatic involvement on clinical evaluation, and were enrolled within 6 months of their initial diagnosis (Figure S1).

Participants completed surveys at the time of enrolment and 6, 12, 36, and 60 months after enrolment. Trained personnel performed chart abstraction 1 year after patient enrolment to collect relevant clinical and treatment information. Men were excluded from the analytic cohort if they were missing data on BMI or if their primary treatment was not RP, RT, or AS. Institutional review board approval was obtained from each site and from Vanderbilt University Medical Center. Participants provided informed consent.

### 2.2 Exposure

We utilized patient‐reported BMI at 3 years, the earliest measurement in CEASAR of this variable. It is unlikely that participants were misclassified by measuring BMI at 3 years rather than baseline because BMI is stable over this timeframe,^17^, ^18^, ^19^ and significant cancer‐related weight change in this population over 3 years is highly unlikely.^20^, ^21^ BMI was treated as a continuous variable in the analysis of BMI and management option and as a binary categorical variable (obese [BMI ≥ 30 kg/m^2^ vs. nonobese BMI < 30 kg/m^2^]) in the analysis of ds‐QoL.

### 2.3 Covariates

Covariates included baseline EPIC‐26 domain scores (i.e., urinary irritative, urinary incontinence, bowel function, sexual function and hormonal function domain scores; continuous), baseline SF‐36 functional scores (physical function, emotional well‐being and energy and fatigue scores; continuous) and the following baseline demographic and clinical features: age at diagnosis, race/ethnicity (White, Black, Hispanic, Asian, other), education (less than high school, high school graduate, same college, college graduate, graduate/professional school), marital status (not married, married), annual income (less than $30 000, $30 001–$50 000, $50 001–$100 000, more than $100000), health insurance type (Medicare, private, Veteran Affairs or military, Medicaid, other, or none), employment (full‐time, part‐time, retired, unemployed), accrual site (Utah, Atlanta, LA, Louisiana, NJ, CaPSURE), total illness burden index for prostate cancer comorbidity score (TIBI‐CaP) (0–2, 3–4, 5, or more), PSA at diagnosis (continuous), clinical tumour stage (T1, T2), biopsy Gleason score (6 or less, 3 + 4, 4 + 3, 8, 9, 10), social support (continuous), participatory decision‐making scale (continuous), Center for Epidemiologic Studies Depression scale (continuous).

### 2.4 Outcomes

We considered two key outcomes. First, we examined the association between BMI and management option (RP, RT, and AS), determined by the combined information from 1‐year chart abstraction, patient‐reported survey, and SEER registry. Second, we examined the association between BMI and ds‐QoL, measured at baseline, 6, 12, 36, and 60 months after enrolment using the previously validated 26‐item Expanded Prostate Index Composite (EPIC‐26).^22^ General health‐related functional outcomes, measured using the Short Form Health Survey (SF‐36),^23^ were explored as secondary outcomes. Domain scores were normalized to a range of 0 to 100, with higher scores representing better function. Results were interpreted according to previously determined minimum clinically important differences (MCID) for each functional domain (4 points for bowel and hormonal, 5 for urinary irritative, 6 for urinary incontinence, 10 for sexual function, 6 for emotional well‐being, 7 for physical functioning, and 9 for energy and fatigue).^24^, ^25^

### 2.5 Statistical analysis

Clinicopathologic and sociodemographic characteristics were summarized by World Health Organization (WHO) BMI category (normal or underweight [BMI < 25 kg/m^2^], overweight [BMI ≥ 25 kg/m^2^ and BMI < 30 kg/m^2^], and obese [BMI ≥ 30 kg/m^2^]) and compared using the Kruskal‐Wallis and chi‐squared tests for continuous and categorical variables, respectively.

Multivariable multinomial logistic regression was used to investigate the association between BMI and management option, and adjusted relative risk ratios (aRRR) of receiving RP and AS compared with RT were estimated and reported with 95% confidence intervals. BMI was treated as a continuous variable in this model, and restricted cubic splines with 3 knots were used for BMI to allow nonlinear association with the outcome. This model included the covariates defined above, and an interaction term for BMI and NCCPCa risk‐category (low to favourable‐intermediate vs. unfavourable‐intermediate to high) was included in the analysis of treatment option (i.e., surgery vs. radiation). As the interaction was determined to not be significant, it was excluded from the final model.

To estimate the association between BMI and ds‐QoL domain scores (i.e., EPIC‐26 and SF‐36 domain scores), we fit multivariable longitudinal linear regression models. In these models, BMI was dichotomized as obese vs. nonobese (BMI ≥ 30 vs. BMI < 30) according to WHO criteria to facilitate presentation of data. A sensitivity analysis for the ds‐QoL analysis was performed using WHO BMI categories of obese (BMI ≥ 30), overweight (BMI ≥ 25 and < 30), and normal or underweight (BMI < 25) and are presented in the supplement (Tables S1 and S2). This demonstrated similar ds‐QoL outcomes in overweight and normal/underweight men. To account for the potential correlation among the outcomes collected longitudinally on the same subject at 6, 12, 36, and 60 months, generalized estimating equations were used with the Huber‐White method to estimate robust covariance matrix. Other covariates in the models were management option (RT, RP, AS), age (continuous, restricted cubic splines with three knots), corresponding baseline EPIC‐26 and SF‐36 domain scores (continuous, restricted cubic splines with three knots), time since treatment (continuous, restricted cubic splines with three knots). SF‐36 general health scale (continuous) at baseline was included in the EPIC‐26 models. We included the same baseline demographic and clinical features as the management option‐BMI model described in the previous paragraph. Finally, interaction terms for BMI and management modality, modality and time since treatment, and BMI and time since treatment were also included. Adjusted mean domain‐score differences with 95% confidence intervals were reported and interpreted using MCID.

Missing covariate values were imputed using the multiple imputation by chained equations procedure.^26^ Two‐sided *p* values less than 0.05 were considered statistically significant. Analyses and figures were produced using R version 4.0.

## 3 RESULTS

Of 7343 men invited to participate, 3634 refused, 432 did not meet CEASAR inclusion criteria, and 890 did not meet additional inclusion criteria for this analysis (e.g. missing BMI or having received ablation or hormone therapy as initial management). Thus, 2378 study participants were included in the analytical cohort (Figure S1); of these, 1287 (54%) underwent RP, 756 (32%) had RT, and 335 (14%) were under AS (Figure S1). Median age at diagnosis was 64 years (quartiles 59–69) and 1818 (77%) were non‐Hispanic White. Median BMI was 27 kg/m^2^. 582 (24%) patients were normal or underweight (BMI < 25); 1118 (47%) were overweight (BMI ≥ 25 and <30), and 678 (29%) were obese (BMI ≥ 30). Men who were younger, Black, had some‐college education or less, or had higher comorbidity scores were more likely to be obese (all *p* < 0.001). There were no significant differences across BMI groups in PSA at diagnosis, biopsy Gleason score, or clinical stage (Table 1).

**TABLE 1:** Baseline demographic, socioeconomic, and clinical characteristics by BMI category. *N* represents the number of nonmissing values.

	Underweight/normal (<25)	Overweight (≥25 and <30)	Obese (≥30)	Combined	*p* value
	(*N* = 582)	(*N* = 1118)	(*N* = 678)	(*N* = 2378)	
Age at diagnosis, median (quartiles)	66 (60, 72)	64 (58, 69)	63 (58, 68)	64 (59, 69)	<0.001
Race	*N* (%)	*N* (%)	*N* (%)	*N* (%)	
White	433 (75%)	879 (79%)	506 (75%)	1818 (77%)	<0.001
Black	71 (12%)	119 (11%)	107 (16%)	297 (13%)	
Hispanic	29 (5%)	80 (7%)	45 (7%)	154 (7%)	
Asian	39 (7%)	22 (2%)	7 (1%)	68 (3%)	
Other	8 (1%)	14 (1%)	10 (1%)	32 (1%)	
Education					
Less than high school	51 (9%)	83 (8%)	54 (8%)	188 (8%)	<0.001
High school graduate	82 (15%)	221 (21%)	133 (21%)	436 (19%)	
Some college	107 (19%)	230 (22%)	174 (27%)	511 (22%)	
College graduate	145 (26%)	258 (24%)	149 (23%)	552 (24%)	
Graduate/professional	179 (32%)	275 (26%)	131 (20%)	585 (26%)	
Marital status					
Not married	122 (22%)	187 (18%)	118 (18%)	427 (19%)	0.11
Married	439 (78%)	879 (82%)	521 (82%)	1839 (81%)	
Income					
<$30 000	113 (21%)	170 (17%)	108 (18%)	391 (18%)	0.12
$30 001–$50 000	83 (16%)	209 (21%)	116 (19%)	408 (19%)	
$50 001–$100 000	160 (30%)	336 (33%)	192 (32%)	688 (32%)	
>$100 000	170 (32%)	301 (30%)	186 (31%)	657 (31%)	
Health insurance					
Medicare	298 (51%)	522 (47%)	287 (42%)	1107 (47%)	0.02
Private/HMO	252 (43%)	545 (49%)	355 (53%)	1152 (49%)	
VA/military/Medicaid/other/none	31 (5%)	50 (4%)	34 (5%)	115 (5%)	
Employment					
Full time	224 (39%)	481 (43%)	309 (46%)	1014 (43%)	0.10
Part time	44 (8%)	100 (9%)	49 (7%)	193 (8%)	
Retired	281 (49%)	482 (43%)	277 (41%)	1040 (44%)	
Unemployed	30 (5%)	46 (4%)	33 (5%)	109 (5%)	
Comorbidity score (TIBI‐CaP)^a^					
0–2	193 (34%)	340 (32%)	132 (20%)	665 (29%)	<0.001
3–4	247 (44%)	439 (41%)	295 (46%)	981 (43%)	
5 or more	126 (22%)	294 (27%)	219 (34%)	639 (28%)	
PSA at diagnosis, corrected, median (IQR)	5 (4, 8)	5 (4, 7)	5 (4, 7)	5 (4, 7)	0.20
Clinical tumour stage					
T1	439 (76%)	856 (77%)	503 (74%)	1798 (76%)	0.48
T2	138 (24%)	260 (23%)	175 (26%)	573 (24%)	
Biopsy Gleason score					
6 or less	297 (51%)	577 (52%)	342 (51%)	1216 (51%)	0.99
3 + 4	164 (28%)	314 (28%)	191 (28%)	669 (28%)	
4 + 3	57 (10%)	115 (10%)	71 (11%)	243 (10%)	
8, 9, or 10	64 (11%)	107 (10%)	69 (10%)	240 (10%)	
Enrolment site					
Utah	58 (10%)	96 (9%)	77 (11%)	231 (10%)	<0.001
Atlanta	92 (16%)	161 (14%)	108 (16%)	361 (15%)	
Los Angeles	186 (32%)	298 (27%)	160 (24%)	644 (27%)	
Louisiana	119 (20%)	281 (25%)	210 (31%)	610 (26%)	
NJ	93 (16%)	207 (19%)	95 (14%)	395 (17%)	
CaPSURE	34 (6%)	75 (7%)	28 (4%)	137 (6%)	
Management option					
Surgery	304 (52%)	611 (55%)	372 (55%)	1287 (54%)	0.33
Radiation therapy	182 (31%)	352 (31%)	222 (33%)	756 (32%)	
Active surveillance	96 (16%)	155 (14%)	84 (12%)	335 (14%)	
Behavioural and Social Interaction Scores, median (quartiles)					
Participatory decision‐making^b^	86 (68, 93)	82 (71, 93)	86 (71, 93)	82 (71, 93)	0.93
Social support^c^	95 (65, 100)	95 (75, 100)	95 (70, 100)	95 (70, 100)	0.07
Depression Scale (CESD)^d^	15 (4, 26)	11 (4, 30)	15 (4, 30)	15 (4, 30)	0.08
Baseline General Health‐Related Quality of Life (SF‐36 domain scores), median (quartiles)^e^					
General health	80 (60, 100)	80 (60, 80)	80 (60, 80)	80 (60, 80)	<0.001
Physical function	100 (85, 100)	95 (85, 100)	90 (70, 100)	95 (85, 100)	<0.001
Emotional well‐being	84 (72, 92)	84 (72, 92)	84 (68, 92)	84 (72, 92)	0.12
Energy and fatigue	80 (65, 90)	75 (65, 85)	70 (55, 85)	75 (60, 85)	<0.001
Baseline Disease‐Specific Function (EPIC‐26 domain scores), median (quartiles)^f^					
Urinary irritative	88 (75, 94)	88 (75, 100)	88 (75, 100)	88 (75, 100)	0.55
Urinary incontinence	100 (85, 100)	100 (85, 100)	100 (79, 100)	100 (85, 100)	0.05
Bowel function	100 (92, 100)	100 (96, 100)	100 (92, 100)	100 (95, 100)	0.48
Sexual function	75 (41, 90)	75 (38, 90)	68 (32, 85)	75 (38, 90)	0.01
Hormonal function	95 (90, 100)	95 (85, 100)	90 (80, 100)	95 (85, 100)	<0.001

^a^ Based on the Total Illness Burden Index for Prostate Cancer (higher scores reflect greater severity and number of comorbid conditions).

^b^ Responses to seven items comprise a score of 0 to 100, where higher scores reflect increased patient choice, control, and responsibility.

^c^ From the Medical Outcomes Study Social Support Scale, five questions are used to create this domain score. Scores are normalized to range from 0 to 100; higher scores reflect greater support.

^d^ Center for Epidemiologic Studies Depression Scale is derived from a 10‐item survey. Scores range from 0 to 100. Higher scores reflect more severe depressive symptoms.

^e^ Medical Outcomes Short Form Health Survey (SF‐36) includes three domain scores, scored from 0 to 100, where higher scores reflect better functioning or less disability. Domain scores are weighted sums from 10, 5, and 4 items for physical functions, emotional well‐being, and energy and fatigue, respectively.

^f^ 26‐item Expanded Prostate Index Composite (EPIC‐26) scores range from 0 to 100. Higher scores reflect better function.

### 3.1 Management Option

**FIGURE 1:**
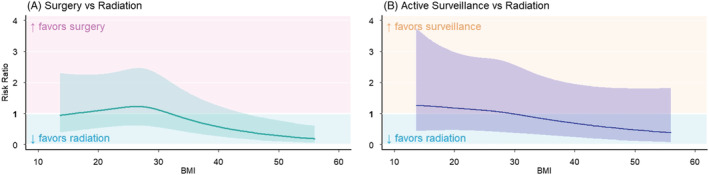
Adjusted risk ratios of receiving (A) RP or (B) AS compared with RT by BMI. Adjusted for demographics, clinicopathologic characteristics, and baseline domain scores. The *y* value of the line represents the adjusted risk ratio of receiving (A) RP or (B) AS compared with RT for a given BMI value. Shading about the line represents the 95% confidence interval of the adjusted risk ratio. Backdrop pink and yellow shading reflect an increased likelihood of (A) RP or (B) AS, respectively, whereas *y* values within the light blue shaded regions represent an increased likelihood of receiving RT.

Primary management option was similar across BMI categories, with 52–55% receiving RP, 31–33% receiving RT, and 12–16% receiving AS in each group (Table 1). To assess for possible effect modification by risk‐group, we included an interaction between BMI and prostate cancer risk group and found no evidence of interaction (*p* = 0.48). The logistic regression models showed that the likelihood of receiving RP compared with RT increased as BMI increased from 15 to 28 kg/m^2^ (Figure 1A). Further increases in BMI were inversely associated with the likelihood of receiving RP compared with RT (Figure 1A), with statistically significant difference for patients with BMI ≥ 33 kg/m^2^ (maximum aRRR = 0.80 [95% CI 0.67 to 0.95], *p* = 0.013 for BMI ≥ 33 vs. BMI = 25) (Table 2). Conversely, the likelihood of receiving AS, compared with RT, exhibited a relatively linear inverse association although the results were not statistically significant at any point across the BMI range (Table 2, Figure 1B).

**TABLE 2:** Adjusted relative risk ratios for receiving RP or AS compared with RT by BMI

BMI at 3rd year (kg/m^2^)	Radical prostatectomy			Active surveillance		
	aRRR	95% CI	*p* value	aRRR	95% CI	*p* value
25	1.00 (referent)			1.00 (referent)		
26	1.01	(0.97, 1.06)	0.64	0.98	(0.93, 1.04)	0.55
30	0.93	(0.81, 1.07)	0.32	0.89	(0.76, 1.06)	0.19
31	0.89	(0.77, 1.03)	0.13	0.87	(0.72, 1.04)	0.13
33	0.8	(0.67, 0.95)	**0.013**	0.81	(0.65, 1.02)	0.076
35	0.71	(0.57, 0.88)	**0.002**	0.76	(0.57, 1.01)	0.062
40	0.53	(0.37, 0.75)	**<0.001**	0.64	(0.40, 1.03)	0.067
45	0.39	(0.24, 0.64)	**<0.001**	0.54	(0.27, 1.07)	0.075

*Note*: Adjusted for demographics, clinicopathologic characteristics, including factors determining prostate cancer risk group classification, and baseline domain scores. aRRR is estimated for receiving each treatment compared with radiation therapy. Statistically significant values are in bold. (e.g., For a man with a BMI of 30, compared with a man with a BMI of 25, the aRRR of receiving RP compared with RT is 0.93) aRRR: adjusted relative risk ratio; RP: radical prostatectomy.

### 3.2 Disease‐Specific Quality of Life Outcomes

There were no statistically significant associations between BMI and baseline patient‐reported urinary irritative, urinary incontinence, and bowel function domain scores. Obese men had significantly lower baseline sexual (*p* = 0.01) and hormonal (*p* < 0.001) domain scores compared with overweight and normal/underweight men (Table 1), while only the difference in hormonal function exceeded the MCID. Based on previous studies demonstrating differential effect of treatment option on ds‐QoL, we stratified our analysis by management option.^2^

**FIGURE 2:**
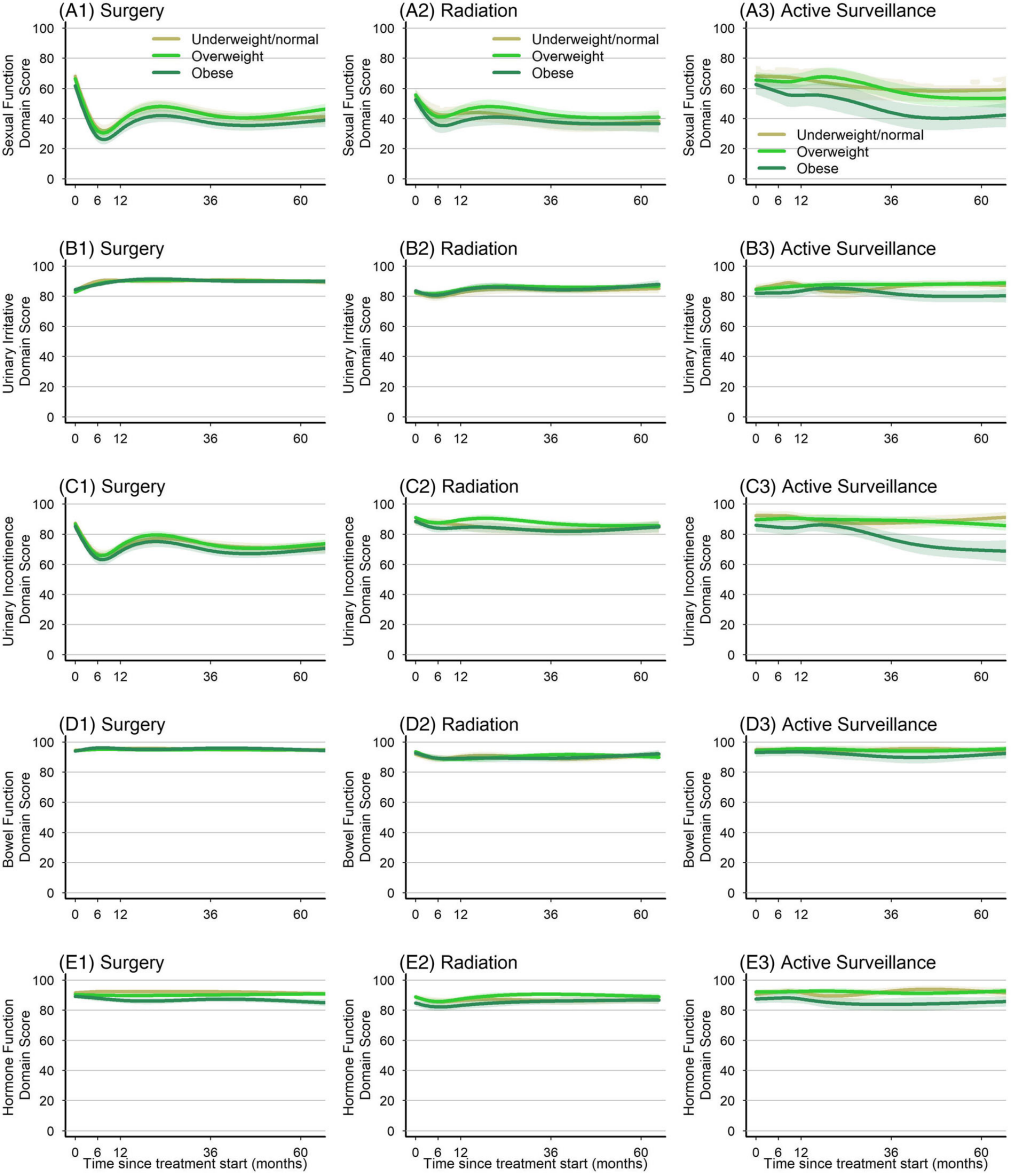
Unadjusted disease‐specific quality‐of‐life outcomes: EPIC‐26 domain scores (estimated means) by WHO BMI category over time, stratified by management option on an intention‐to‐treat basis. Sexual, urinary irritative, urinary incontinence, bowel, and hormonal function domain scores are derived from the 26‐item Expanded Prostate Cancer Index Composite (ranges from 0 to 100; higher scores represent better function or less disability). The curves indicate the unadjusted estimated mean domain score. Faint shading about the curves represents the 95% confidence interval.

**FIGURE 3:**
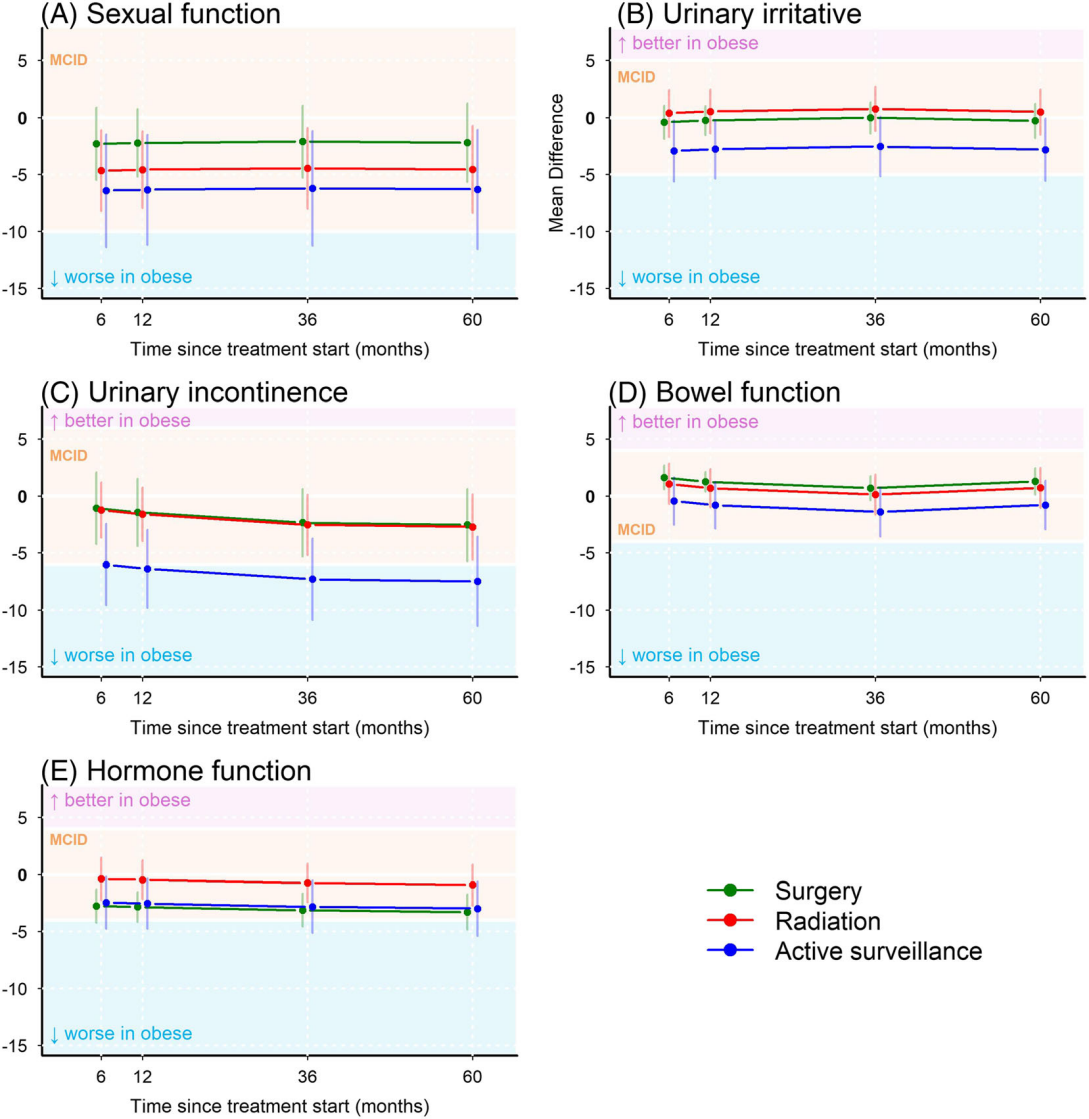
Adjusted EPIC‐26 domain score mean‐differences between obese and nonobese men, stratified by management option, over time. EPIC‐26 domain score adjusted mean differences between obese and nonobese men are represented by the points and curves, which are colourized to reflect management option. Whiskers represent the 95% confidence intervals about each adjusted mean difference. Each graph is shaded so that curves residing in yellow shading represent domain score differences less than the threshold of previously validated clinically important differences. If obese men had worse function in a particular domain, the curve resides in the blue shading. They reside in the pink shading if obese men had better function in a given domain.

Unadjusted results (Figure 2) are presented to provide a basis for interpretation of the *adjusted* mean differences between obese and nonobese men in each treatment group (Figure 3). After stratifying by management option and adjusting for baseline function and other covariates, we found no differences between obese and nonobese men through 5 years in EPIC‐26 functional domain scores that exceeded the MCID, excepting obese men who received active surveillance, who had worse urinary incontinence by 6 months and through 5 years (Figure 3). However, this difference attenuated and was no longer significant when the analysis was restricted to men who remained on AS without subsequent definitive therapy (i.e., as‐treated) (Figure S2). Although there were statistically significant associations between obesity and sexual function in men who received RT and AS at all time points, none exceeded the MCID threshold. Statistically significant differences in bowel and hormonal function in obese men who received RP also did not exceed the MCID.

As the use of ADT may also have a differential impact on obese and nonobese men,^27^ we did further analyses within the RT group, stratifying by ADT use. Obese men receiving RT with ADT had worse hormone function by 6 months through 5 years, exceeding the MCID, compared with nonobese men (effect size range: −4.23 to −4.66 points), while no significant differences were observed between obese and nonobese men who received RT‐alone. In the RT + ADT group, obesity was also associated with significantly worse sexual function, but this did not exceed MCID (Table S3).

Obesity was not an independent predictor of general health‐related QoL (SF‐36) when stratified by management option (Table S4).

## 4 DISCUSSION

In this population‐based, prospective cohort of men with localized prostate cancer, we found that obese men with BMI ≥ 33 kg/m^2^ were less likely to undergo RP compared with RT. We did not find an association between obesity and ds‐QoL outcomes in men treated with definitive local therapy. Conversely, among men who started on AS, obese men had worse urinary incontinence than nonobese men after controlling for covariates, which attenuated and was no longer significant in an as‐treated sensitivity analysis.

To our knowledge, only one other study has assessed the association between BMI and management option in localized prostate cancer. The results of that study similarly demonstrated that increasing BMI beyond a certain threshold is associated with increased likelihood of receiving nonsurgical management (including AS) compared with RP, which was most pronounced in men with BMI ≥ 35 kg/m^2^.^28^ This may be due to known associations between obesity and surgical complications,^29^ or urologists may counsel obese men to pursue treatments other than RP for fear of respiratory insufficiency when patients are in steep Trendelenburg during RARP.^6^ This trend may also be due to surgeon perception of worse functional outcomes in obese men undergoing RP than nonobese men, which this study addresses.

In this study, we did not find clinically meaningful differences in patient‐reported functional outcomes between obese and nonobese men undergoing definitive treatment. This is consistent with several previous studies.^5^, ^9^, ^12^ However, most of these studies had limited follow‐up and focused on older surgical techniques. Research assessing the relationship between obesity and ds‐QoL was even more scarce among men managed with RT or AS.^13^

Though some studies have demonstrated worse urinary‐ and sexual‐ function in obese men who underwent RP, the larger studies were retrospective or failed to control for baseline function. The few prospective studies were small, single‐center studies.^4^, ^30^ For example, Ahlering et al. investigated 100 men status‐post RARP and found that obesity was associated with worse pad‐free continence at 6 months. A meta‐analysis of 13 studies found obese men had worse impotence (OR = 1.29, 95% CI 1.03–1.61, *p* = 0.02) and incontinence (OR = 1.41, 95% CI 1.13–1.77, *p* = 0.003) than nonobese men 1 year after RARP.^30^ Evidence from CaPSURE (*n* = 672), corroborates our results, finding no association between obesity and urinary or sexual ds‐QoL after RP.^31^

Among untreated men on AS, there were no differences in ds‐QoL scores by BMI. The finding of worse urinary incontinence in obese men on AS in our study was likely due to secondary effects of treatment. Among men initially managed with AS, obese men were more likely to undergo definitive therapy than nonobese men (46% vs. 26%) (Figure S2).

Our study's strengths include utilization of previously validated, patient‐reported ds‐QoL surveys (EPIC‐26 and SF‐36), large sample size, multivariable models controlling for baseline function, and the use of a population composed of men receiving contemporary guideline‐recommended management options, including RP (≥68% RARP), RT, and AS. There are several limitations. First, the study, like any nonrandomized observational cohort is subject to confounding by indication and unmeasured covariates. This would at least include RP factors like nerve‐sparing‐, bladder neck preservation‐ and Retzius‐space sparing‐ techniques, which are associated with functional outcomes.^32^, ^33^, ^34^ Selection bias at the level of the counselling provider is also suggested by the number of patients on AS who were incontinent at baseline, and since 2010, there has been an increase in the fraction of men with low‐risk PCa receiving AS.^35^ These limitations are mitigated by careful data collection, use of multivariable analysis controlling for known confounders, and interpretation of results according to previously validated minimum clinically important differences.

A major limitation of this study is the initial measurement of BMI in the third year. Initial surveys captured limited information to mitigate respondent burden and maximize response rates. Despite this, there exists no compelling evidence that men newly diagnosed with localized PCa experience significant changes in their BMI.^20^, ^21^ For example, data from the ProtecT trial showed no significant difference in men's pre‐ and post‐diagnosis BMI (9‐month follow‐up, *p* = 0.32). Another study found a statistically significant difference in the BMI of men with PCa after a mean follow‐up of 2.0‐years, but it was from 26.1 to 26.3 kg/m^2^ (*ß* = +0.28 kg/m^2^, 95% CI [0.00–0.55]). Importantly, misclassification bias from possibly undetected increase in BMI would represent crossover and increase the likelihood of a type II error.

## 5 CONCLUSIONS

In this population‐based, prospective cohort of men with localized prostate cancer, we found that, above BMI of 28 kg/m^2^, further increases in BMI were inversely associated with the likelihood of surgical treatment compared with RT. While obesity may contribute to treatment choice, among those receiving definitive treatment, we found no evidence of worse functional outcomes in obese men compared with nonobese men undergoing definitive treatment, nor in men who remained on AS. This information may be valuable to physicians and patients considering management options for localized prostate cancer and may expand management options for obese men.

## CONFLICT OF INTEREST

The authors have no competing interests.

## AUTHOR CONTRIBUTIONS

Research conceptualization: all authors; Data acquisition: MC, MG, AH, MH, BO, LEP, AS, and XCW; Data curation: LCH, ZZ, TK; Data Analysis: NLS, CJW, LCH, JET, ZZ, KF, AM, TK, DAB; Funding acquisition: DFP, DAB; Methodology: NLS, CJW, LCH, JET, ZZ, SG, SK, TK, DAB; Writing—original draft: NLS, CJW, JT, TK, DAB; Writing—review and editing: all authors.

## Supporting information


**Table S1** Adjusted disease‐specific functional outcomes: EPIC‐26 domain scores by WHO BMI Category, stratified by management option, adjusted for patient demographic, tumor, and baseline functional characteristics.

**Table S2** Health‐Related Quality of Life Outcomes: SF‐36 domain scores by WHO BMI Category, stratified by management option, adjusted for patient demographic, tumor, and baseline functional characteristics.

**Table S3** Disease‐Specific Quality‐of‐Life outcomes: EPIC‐26 domain scores by obesity status, stratified by management option and receipt of ADT, effect size adjusted for patient demographic, tumor, and baseline functional characteristics.

**Table S4** General health‐related quality of life outcomes: SF‐36 domain scores by obesity status, stratified by management option, adjusted for patient demographic, tumor, and baseline functional characteristics.

**Table S5** Internal cohort BMI stability from 3 years to 5 years after baseline

**Table S6** Multinomial multivariable logistic regression model for the outcome of management option including all covariates.

**Figure S1:** Diagram of the assembly of the Comparative Effectiveness Analysis of Surgery and Radiation (CEASAR) study cohort and the final analytic cohort

**Figure S2:** Adjusted EPIC‐26 domain score mean‐differences between obese and non‐obese men, stratified by management option, over time in an as‐treated analysis (i.e. excluding men initially on AS who underwent later definitive treatment)
