# Supplementary material for: Association between body mass index and localized prostate cancer management and disease‐specific quality of life
Source: BJUI Compass. 2022 Nov 2;4(2):223–33. doi: 10.1002/bco2.197 (PMC9931544; doi:10.1002/bco2.197)
Supplement: Supplementary file 1 — Table S1 Adjusted disease‐specific functional outcomes: EPIC‐26 domain scores by WHO BMI Category, stratified by management option, adjusted for patient demographic, tumor, and baseline functional characteristics. [file BCO2-4-223-s007.docx]

Supplemental Table 1: Adjusted disease-specific functional outcomes: EPIC-26 domain scores by WHO BMI Category, stratified by management option, adjusted for patient demographic, tumor, and baseline functional characteristics.

|  |  |  | Obese vs Underweight/normal | | | Overweight vs Underweight/normal | | | Obese vs Overweight | | |
| --- | --- | --- | --- | --- | --- | --- | --- | --- | --- | --- | --- |
| Treatment | Month | N | Effect Size | 95% CI | P-value | Effect Size | 95% CI | P- value | Effect Size | 95% CI | P-value |
| **Urinary Irritative** | | | | | | | | | | | |
| Surgery | 6 | 1210 | -1.11 | (-2.83, 0.62) | 0.21 | -1.07 | (-2.55, 0.42) | 0.16 | -0.04 | (-1.61, 1.53) | 0.96 |
|  | 12 | 1210 | -0.51 | (-2.02, 1.00) | 0.51 | -0.41 | (-1.70, 0.89) | 0.54 | -0.1 | (-1.46, 1.26) | 0.88 |
|  | 36 | 1264 | 0.46 | (-1.22, 2.13) | 0.59 | 0.7 | (-0.76, 2.16) | 0.35 | -0.24 | (-1.71, 1.23) | 0.75 |
|  | 60 | 1100 | -0.33 | (-2.18, 1.51) | 0.73 | -0.08 | (-1.71, 1.55) | 0.92 | -0.25 | (-1.84, 1.34) | 0.76 |
| Radiation | 6 | 713 | 0.48 | (-2.10, 3.05) | 0.72 | 0.13 | (-2.06, 2.31) | 0.91 | 0.35 | (-1.83, 2.53) | 0.75 |
|  | 12 | 713 | 1.07 | (-1.34, 3.48) | 0.38 | 0.79 | (-1.25, 2.82) | 0.45 | 0.29 | (-1.74, 2.31) | 0.78 |
|  | 36 | 735 | 2.04 | (-0.41, 4.49) | 0.1 | 1.9 | (-0.17, 3.97) | 0.07 | 0.14 | (-1.90, 2.19) | 0.89 |
|  | 60 | 618 | 1.25 | (-1.30, 3.79) | 0.34 | 1.11 | (-1.07, 3.29) | 0.32 | 0.14 | (-1.95, 2.22) | 0.90 |
| Active surveillance | 6 | 317 | -3.04 | (-6.14, 0.05) | 0.05 | -0.12 | (-2.46, 2.22) | 0.92 | -2.93 | (-5.73, -0.12) | 0.04 |
|  | 12 | 315 | -2.45 | (-5.41, 0.52) | 0.11 | 0.54 | (-1.68, 2.76) | 0.64 | -2.99 | (-5.67, -0.31) | 0.03 |
|  | 36 | 326 | -1.48 | (-4.51, 1.55) | 0.34 | 1.65 | (-0.66, 3.96) | 0.16 | -3.13 | (-5.86, -0.40) | 0.03 |
|  | 60 | 280 | -2.27 | (-5.46, 0.92) | 0.16 | 0.87 | (-1.54, 3.27) | 0.48 | -3.14 | (-5.97, -0.30) | 0.03 |
| **Urinary incontinence** | | | | | | | | | | | |
| Surgery | 6 | 1226 | -1.19 | (-5.13, 2.76) | 0.56 | -0.24 | (-3.68, 3.20) | 0.89 | -0.95 | (-4.28, 2.39) | 0.58 |
|  | 12 | 1184 | -0.86 | (-4.59, 2.87) | 0.65 | 0.82 | (-2.42, 4.06) | 0.62 | -1.68 | (-4.81, 1.45) | 0.29 |
|  | 36 | 1259 | -0.85 | (-4.58, 2.88) | 0.65 | 2.22 | (-1.06, 5.50) | 0.19 | -3.07 | (-6.20, 0.06) | 0.06 |
|  | 60 | 1103 | -2.44 | (-6.44, 1.55) | 0.23 | 0.09 | (-3.42, 3.60) | 0.96 | -2.53 | (-5.89, 0.82) | 0.14 |
| Radiation | 6 | 720 | -1.67 | (-4.78, 1.45) | 0.3 | -0.69 | (-3.39, 2.02) | 0.62 | -0.98 | (-3.53, 1.57) | 0.45 |
|  | 12 | 691 | -1.34 | (-4.36, 1.68) | 0.38 | 0.37 | (-2.19, 2.93) | 0.78 | -1.71 | (-4.15, 0.72) | 0.17 |
|  | 36 | 735 | -1.33 | (-4.71, 2.04) | 0.44 | 1.77 | (-1.06, 4.61) | 0.22 | -3.1 | (-5.86, -0.35) | 0.03 |
|  | 60 | 618 | -2.92 | (-6.55, 0.70) | 0.11 | -0.36 | (-3.43, 2.72) | 0.82 | -2.57 | (-5.57, 0.43) | 0.09 |
| Active surveillance | 6 | 322 | -5.76 | (-9.89, -1.63) | 0.01 | 0.4 | (-2.76, 3.56) | 0.8 | -6.16 | (-9.88, -2.45) | <0.01 |
|  | 12 | 310 | -5.44 | (-9.41, -1.46) | 0.01 | 1.46 | (-1.54, 4.46) | 0.34 | -6.9 | (-10.46, -3.34) | <0.001 |
|  | 36 | 325 | -5.43 | (-9.58, -1.28) | 0.01 | 2.86 | (-0.28, 6.00) | 0.07 | -8.29 | (-11.99, -4.58) | <0.001 |
|  | 60 | 282 | -7.02 | (-11.53, -2.50) | 0 | 0.73 | (-2.59, 4.06) | 0.67 | -7.75 | (-11.81, -3.68) | <0.001 |
| **Sexual function** | | | | | | | | | | | |
| Surgery | 6 | 1204 | -2.8 | (-6.93, 1.32) | 0.18 | -0.81 | (-4.59, 2.98) | 0.68 | -2 | (-5.39, 1.39) | 0.25 |
|  | 12 | 1210 | -2.25 | (-6.11, 1.61) | 0.25 | -0.08 | (-3.63, 3.47) | 0.97 | -2.17 | (-5.34, 1.00) | 0.18 |
|  | 36 | 1256 | -0.95 | (-5.04, 3.14) | 0.65 | 1.7 | (-2.12, 5.52) | 0.38 | -2.65 | (-6.06, 0.77) | 0.13 |
|  | 60 | 1098 | -0.8 | (-5.20, 3.60) | 0.72 | 2.08 | (-1.87, 6.03) | 0.3 | -2.88 | (-6.56, 0.80) | 0.13 |
| Radiation | 6 | 679 | -6.26 | (-10.81, -1.71) | 0.01 | -2.46 | (-6.54, 1.62) | 0.24 | -3.8 | (-7.58, -0.01) | 0.05 |
|  | 12 | 698 | -5.7 | (-10.03, -1.37) | 0.01 | -1.74 | (-5.61, 2.13) | 0.38 | -3.96 | (-7.53, -0.40) | 0.03 |
|  | 36 | 710 | -4.4 | (-8.94, 0.14) | 0.06 | 0.04 | (-4.06, 4.14) | 0.99 | -4.44 | (-8.23, -0.66) | 0.02 |
|  | 60 | 592 | -4.25 | (-9.03, 0.52) | 0.08 | 0.42 | (-3.75, 4.59) | 0.84 | -4.67 | (-8.74, -0.61) | 0.02 |
| Active surveillance | 6 | 307 | -5.98 | (-11.93, -0.04) | 0.05 | 0.66 | (-4.35, 5.68) | 0.8 | -6.65 | (-11.89, -1.40) | 0.01 |
|  | 12 | 298 | -5.43 | (-11.21, 0.35) | 0.07 | 1.39 | (-3.40, 6.18) | 0.57 | -6.82 | (-11.93, -1.71) | 0.01 |
|  | 36 | 316 | -4.13 | (-10.12, 1.86) | 0.18 | 3.17 | (-1.80, 8.13) | 0.21 | -7.3 | (-12.60, -1.99) | 0.01 |
|  | 60 | 266 | -3.98 | (-10.27, 2.31) | 0.22 | 3.55 | (-1.70, 8.80) | 0.19 | -7.53 | (-13.05, -2.01) | 0.01 |
| **Bowel function** | | | | | | | | | | | |
| Surgery | 6 | 1232 | 1.42 | (0.08, 2.77) | 0.04 | -0.33 | (-1.58, 0.92) | 0.61 | 1.75 | (0.62, 2.89) | <0.01 |
|  | 12 | 1223 | 1.29 | (0.15, 2.43) | 0.03 | 0.04 | (-1.01, 1.09) | 0.94 | 1.25 | (0.31, 2.19) | 0.01 |
|  | 36 | 1278 | 1.15 | (-0.23, 2.53) | 0.1 | 0.69 | (-0.61, 1.98) | 0.3 | 0.46 | (-0.66, 1.58) | 0.42 |
|  | 60 | 1114 | 1.49 | (-0.05, 3.03) | 0.06 | 0.3 | (-1.10, 1.70) | 0.68 | 1.19 | (-0.04, 2.43) | 0.06 |
| Radiation | 6 | 718 | -0.06 | (-2.18, 2.05) | 0.95 | -1.71 | (-3.61, 0.19) | 0.08 | 1.64 | (-0.26, 3.55) | 0.09 |
|  | 12 | 723 | -0.2 | (-2.19, 1.80) | 0.85 | -1.34 | (-3.08, 0.40) | 0.13 | 1.14 | (-0.66, 2.95) | 0.22 |
|  | 36 | 751 | -0.34 | (-2.47, 1.78) | 0.75 | -0.7 | (-2.52, 1.13) | 0.46 | 0.35 | (-1.53, 2.24) | 0.71 |
|  | 60 | 622 | 0 | (-2.18, 2.18) | 1 | -1.09 | (-3.01, 0.84) | 0.27 | 1.09 | (-0.78, 2.96) | 0.26 |
| Active surveillance | 6 | 324 | -0.31 | (-2.72, 2.11) | 0.8 | 0.22 | (-1.61, 2.05) | 0.81 | -0.53 | (-2.74, 1.68) | 0.64 |
|  | 12 | 318 | -0.44 | (-2.75, 1.87) | 0.71 | 0.59 | (-1.09, 2.27) | 0.49 | -1.03 | (-3.18, 1.12) | 0.35 |
|  | 36 | 335 | -0.58 | (-2.99, 1.82) | 0.63 | 1.24 | (-0.54, 3.01) | 0.17 | -1.82 | (-4.09, 0.45) | 0.12 |
|  | 60 | 282 | -0.24 | (-2.67, 2.19) | 0.85 | 0.85 | (-1.00, 2.69) | 0.37 | -1.09 | (-3.33, 1.16) | 0.34 |
| **Hormone function** | | | | | | | | | | | |
| Surgery | 6 | 1212 | -4.15 | (-5.82, -2.47) | <0.001 | -2.05 | (-3.38, -0.72) | 0 | -2.1 | (-3.65, -0.55) | 0.01 |
|  | 12 | 1211 | -3.92 | (-5.42, -2.42) | <0.001 | -1.57 | (-2.76, -0.38) | 0.01 | -2.35 | (-3.75, -0.96) | <0.001 |
|  | 36 | 1263 | -3.48 | (-5.16, -1.80) | <0.001 | -0.49 | (-1.87, 0.89) | 0.49 | -2.99 | (-4.53, -1.46) | <0.001 |
|  | 60 | 1105 | -3.64 | (-5.51, -1.77) | <0.001 | -0.48 | (-1.99, 1.03) | 0.53 | -3.16 | (-4.77, -1.54) | <0.001 |
| Radiation | 6 | 704 | -0.43 | (-2.78, 1.93) | 0.72 | -0.04 | (-2.00, 1.92) | 0.97 | -0.38 | (-2.34, 1.57) | 0.7 |
|  | 12 | 708 | -0.2 | (-2.38, 1.99) | 0.86 | 0.44 | (-1.40, 2.28) | 0.64 | -0.64 | (-2.42, 1.14) | 0.48 |
|  | 36 | 736 | 0.24 | (-1.98, 2.46) | 0.83 | 1.52 | (-0.40, 3.43) | 0.12 | -1.28 | (-3.05, 0.50) | 0.16 |
|  | 60 | 611 | 0.08 | (-2.29, 2.46) | 0.94 | 1.53 | (-0.51, 3.57) | 0.14 | -1.44 | (-3.31, 0.42) | 0.13 |
| Active surveillance | 6 | 320 | -3.5 | (-6.10, -0.90) | 0.01 | -1.55 | (-3.42, 0.33) | 0.11 | -1.95 | (-4.33, 0.42) | 0.11 |
|  | 12 | 314 | -3.27 | (-5.76, -0.79) | 0.01 | -1.06 | (-2.80, 0.67) | 0.23 | -2.21 | (-4.49, 0.07) | 0.06 |
|  | 36 | 331 | -2.83 | (-5.42, -0.24) | 0.03 | 0.01 | (-1.80, 1.83) | 0.99 | -2.85 | (-5.24, -0.46) | 0.02 |
|  | 60 | 280 | -2.99 | (-5.75, -0.23) | 0.03 | 0.03 | (-1.97, 2.02) | 0.98 | -3.02 | (-5.49, -0.54) | 0.02 |
