# Supplementary material for: Association between body mass index and localized prostate cancer management and disease‐specific quality of life
Source: BJUI Compass. 2022 Nov 2;4(2):223–33. doi: 10.1002/bco2.197 (PMC9931544; doi:10.1002/bco2.197)
Supplement: Supplementary file 2 — Table S2 Health‐Related Quality of Life Outcomes: SF‐36 domain scores by WHO BMI Category, stratified by management option, adjusted for patient demographic, tumor, and baseline functional characteristics. [file BCO2-4-223-s006.docx]

Supplemental Table 2: Health-Related Quality of Life Outcomes: SF-36 domain scores by WHO BMI Category, stratified by management option, adjusted for patient demographic, tumor, and baseline functional characteristics.

|  | | Obese vs Underweight/normal | | | Overweight vs Underweight/normal | | | Obese vs Overweight | | |
| --- | --- | --- | --- | --- | --- | --- | --- | --- | --- | --- |
| Treatment | Month | Effect Size | 95% CI | P-value | Effect Size | 95% CI | P-value | Effect Size | 95% CI | P-value |
| **Physical function** | | | | | | | | | | |
| **Surgery** | 6 | -2.02 | (-4.24, 0.21) | 0.01 | -0.87 | (-2.68, 0.94) | 0.34 | -1.15 | (-3.02, 0.73) | 0.23 |
|  | 12 | -1.38 | (-3.40, 0.64) | 0.18 | -0.45 | (-2.09, 1.20) | 0.60 | -0.93 | (-2.63, 0.77) | 0.28 |
|  | 36 | -1.53 | (-3.76, 0.70) | 0.18 | -0.35 | (-2.24, 1.54) | 0.72 | -1.18 | (-3.10, 0.74) | 0.23 |
|  | 60 | -5.03 | (-7.58, -2.49) | <0.001 | -2.24 | (-4.46, -0.03) | 0.05 | -2.79 | (-4.95, -0.63) | 0.01 |
| **Radiation** | 6 | -3.12 | (-6.02, -0.21) | 0.04 | -0.2 | (-2.73, 2.33) | 0.88 | -2.91 | (-5.38, -0.45) | 0.02 |
|  | 12 | -2.48 | (-5.29, 0.33) | 0.08 | 0.22 | (-2.22, 2.67) | 0.86 | -2.7 | (-5.09, -0.31) | 0.03 |
|  | 36 | -2.63 | (-5.69, 0.44) | 0.09 | 0.32 | (-2.36, 3.00) | 0.82 | -2.94 | (-5.60, -0.29) | 0.03 |
|  | 60 | -6.13 | (-9.40, -2.87) | <0.001 | -1.58 | (-4.51, 1.36) | 0.29 | -4.56 | (-7.35, -1.76) | <0.01 |
| **Active surveillance** | 6 | -4.65 | (-8.45, -0.84) | 0.02 | -0.75 | (-3.59, 2.09) | 0.60 | -3.89 | (-7.63, -0.15) | 0.04 |
|  | 12 | -4.01 | (-7.71, -0.31) | 0.03 | -0.33 | (-3.07, 2.42) | 0.82 | -3.68 | (-7.30, -0.06) | 0.05 |
|  | 36 | -4.16 | (-8.00, -0.31) | 0.03 | -0.23 | (-3.11, 2.65) | 0.88 | -3.92 | (-7.64, -0.21) | 0.04 |
|  | 60 | -7.66 | (-11.70, -3.62) | <0.001 | -2.13 | (-5.18, 0.93) | 0.17 | -5.54 | (-9.50, -1.57) | 0.01 |
| **Emotional wellbeing** | | | | | | | | | | |
| **Surgery** | 6 | -0.26 | (-1.95, 1.43) | 0.76 | -0.81 | (-2.27, 0.66) | 0.28 | 0.55 | (-1.03, 2.12) | 0.50 |
|  | 12 | -0.62 | (-2.16, 0.92) | 0.43 | -0.85 | (-2.18, 0.48) | 0.21 | 0.23 | (-1.22, 1.68) | 0.76 |
|  | 36 | -0.91 | (-2.63, 0.81) | 0.30 | -0.69 | (-2.23, 0.85) | 0.38 | -0.22 | (-1.77, 1.33) | 0.78 |
|  | 60 | 0.24 | (-1.63, 2.11) | 0.80 | -0.14 | (-1.81, 1.53) | 0.87 | 0.38 | (-1.31, 2.06) | 0.66 |
| **Radiation** | 6 | 0.98 | (-1.38, 3.34) | 0.41 | 0.79 | (-1.34, 2.91) | 0.47 | 0.2 | (-1.52, 1.92) | 0.82 |
|  | 12 | 0.62 | (-1.59, 2.83) | 0.58 | 0.75 | (-1.22, 2.72) | 0.46 | -0.12 | (-1.78, 1.53) | 0.88 |
|  | 36 | 0.33 | (-2.02, 2.69) | 0.78 | 0.9 | (-1.17, 2.98) | 0.39 | -0.57 | (-2.39, 1.25) | 0.54 |
|  | 60 | 1.48 | (-1.18, 4.14) | 0.28 | 1.45 | (-0.92, 3.82) | 0.23 | 0.03 | (-1.91, 1.96) | 0.98 |
| **Active surveillance** | 6 | -1.64 | (-4.51, 1.23) | 0.26 | -0.27 | (-2.62, 2.08) | 0.82 | -1.37 | (-3.94, 1.20) | 0.30 |
|  | 12 | -2 | (-4.76, 0.76) | 0.15 | -0.31 | (-2.54, 1.92) | 0.78 | -1.69 | (-4.19, 0.81) | 0.19 |
|  | 36 | -2.29 | (-5.14, 0.56) | 0.12 | -0.15 | (-2.45, 2.15) | 0.90 | -2.14 | (-4.73, 0.46) | 0.11 |
|  | 60 | -1.14 | (-4.17, 1.88) | 0.46 | 0.4 | (-2.06, 2.85) | 0.75 | -1.54 | (-4.24, 1.17) | 0.26 |
| **Energy and fatigue** | | | | | | | | | | |
| **Surgery** | 6 | -2.61 | (-4.69, -0.52) | 0.01 | -1.02 | (-2.81, 0.76) | 0.26 | -1.58 | (-3.51, 0.35) | 0.11 |
|  | 12 | -2.75 | (-4.64, -0.86) | <0.01 | -0.81 | (-2.45, 0.83) | 0.33 | -1.94 | (-3.68, -0.19) | 0.03 |
|  | 36 | -2.87 | (-4.96, -0.77) | 0.01 | -0.29 | (-2.16, 1.59) | 0.76 | -2.58 | (-4.44, -0.72) | 0.01 |
|  | 60 | -2.43 | (-4.72, -0.14) | 0.04 | -0.19 | (-2.21, 1.84) | 0.86 | -2.24 | (-4.30, -0.19) | 0.03 |
| **Radiation** | 6 | -0.97 | (-3.75, 1.80) | 0.49 | 0.49 | (-1.91, 2.88) | 0.69 | -1.46 | (-3.72, 0.81) | 0.21 |
|  | 12 | -1.11 | (-3.79, 1.56) | 0.41 | 0.7 | (-1.58, 2.98) | 0.55 | -1.81 | (-3.96, 0.33) | 0.10 |
|  | 36 | -1.23 | (-4.12, 1.65) | 0.40 | 1.22 | (-1.20, 3.65) | 0.32 | -2.46 | (-4.76, -0.15) | 0.04 |
|  | 60 | -0.8 | (-3.76, 2.16) | 0.60 | 1.32 | (-1.19, 3.84) | 0.30 | -2.12 | (-4.54, 0.29) | 0.08 |
| **Active surveillance** | 6 | -3.08 | (-6.42, 0.26) | 0.07 | -1.62 | (-4.30, 1.05) | 0.24 | -1.46 | (-4.52, 1.60) | 0.35 |
|  | 12 | -3.22 | (-6.41, -0.04) | 0.05 | -1.41 | (-3.94, 1.13) | 0.28 | -1.82 | (-4.75, 1.12) | 0.23 |
|  | 36 | -3.34 | (-6.59, -0.09) | 0.04 | -0.88 | (-3.47, 1.70) | 0.50 | -2.46 | (-5.47, 0.55) | 0.11 |
|  | 60 | -2.91 | (-6.33, 0.52) | 0.10 | -0.78 | (-3.48, 1.91) | 0.57 | -2.12 | (-5.28, 1.03) | 0.19 |
