# Supplementary material for: Association between body mass index and localized prostate cancer management and disease‐specific quality of life
Source: BJUI Compass. 2022 Nov 2;4(2):223–33. doi: 10.1002/bco2.197 (PMC9931544; doi:10.1002/bco2.197)
Supplement: Supplementary file 3 — Table S3 Disease‐Specific Quality‐of‐Life outcomes: EPIC‐26 domain scores by obesity status, stratified by management option and receipt of ADT, effect size adjusted for patient demographic, tumor, and baseline functional characteristics. [file BCO2-4-223-s008.docx]

Supplemental Table 3: Disease-Specific Quality-of-Life outcomes: EPIC-26 domain scores by obesity status, stratified by management option and receipt of ADT, effect size adjusted for patient demographic, tumor, and baseline functional characteristics.

|  |  |  | Unadjusted median (quartiles) | | Obese vs Non-obese | | |
| --- | --- | --- | --- | --- | --- | --- | --- |
| Treatment | Month | N | Obese | Non-obese | Effect | 95% CI | p-value |
| **Urinary Irritative** | | | | | | | |
| Surgery | 0 | 1239 | 94 (75, 100) | 88 (75, 100) |  |  |  |
|  | 6 | 1210 | 94 (81, 100) | 94 (81, 100) | -0.48 | [-1.94, 0.98] | 0.52 |
|  | 12 | 1210 | 94 (81, 100) | 94 (88, 100) | -0.28 | [-1.56, 0.99] | 0.67 |
|  | 36 | 1264 | 94 (88, 100) | 94 (88, 100) | 0.04 | [-1.34, 1.42] | 0.95 |
|  | 60 | 1100 | 94 (88, 100) | 94 (88, 100) | -0.23 | [-1.73, 1.26] | 0.76 |
| Radiation with ADT | 0 | 236 | 88 (75, 94) | 88 (75, 94) |  |  |  |
|  | 6 | 236 | 81 (69, 94) | 81 (69, 94) | -1.05 | [-4.69, 2.58] | 0.57 |
|  | 12 | 232 | 88 (75, 94) | 88 (75, 94) | -0.85 | [-4.41, 2.71] | 0.64 |
|  | 36 | 240 | 88 (75, 97) | 88 (81, 100) | -0.53 | [-4.13, 3.07] | 0.77 |
|  | 60 | 194 | 94 (81, 94) | 88 (81, 100) | -0.8 | [-4.47, 2.87] | 0.67 |
| Radiation without ADT | 0 | 489 | 88 (81, 94) | 88 (75, 100) |  |  |  |
|  | 6 | 472 | 88 (75, 94) | 88 (75, 94) | 0.98 | [-1.37, 3.33] | 0.41 |
|  | 12 | 476 | 88 (75, 94) | 88 (75, 94) | 1.18 | [-1.04, 3.40] | 0.30 |
|  | 36 | 490 | 88 (81, 100) | 88 (77, 94) | 1.5 | [-0.72, 3.72] | 0.19 |
|  | 60 | 419 | 94 (81, 100) | 88 (81, 100) | 1.23 | [-1.00, 3.46] | 0.28 |
| Active surveillance | 0 | 320 | 88 (75, 94) | 88 (75, 100) |  |  |  |
|  | 6 | 317 | 88 (75, 94) | 94 (81, 100) | -2.98 | [-5.66, -0.31] | 0.03 |
|  | 12 | 315 | 88 (81, 94) | 88 (81, 100) | -2.78 | [-5.35, -0.22] | 0.03 |
|  | 36 | 326 | 88 (69, 94) | 94 (81, 100) | -2.46 | [-5.07, 0.15] | 0.07 |
|  | 60 | 280 | 81 (69, 94) | 88 (81, 100) | -2.73 | [-5.46, -0.01] | 0.05 |
| **Urinary incontinence** | | | | | | | |
| Surgery | 0 | 1243 | 100 (77, 100) | 100 (85, 100) |  |  |  |
|  | 6 | 1226 | 67 (44, 94) | 67 (46, 94) | -1.05 | [-4.20, 2.11] | 0.52 |
|  | 12 | 1184 | 73 (52, 100) | 79 (54, 100) | -1.43 | [-4.40, 1.54] | 0.34 |
|  | 36 | 1259 | 73 (52, 92) | 79 (58, 100) | -2.35 | [-5.31, 0.61] | 0.12 |
|  | 60 | 1103 | 73 (52, 94) | 77 (54, 100) | -2.51 | [-5.69, 0.67] | 0.12 |
| Radiation with ADT | 0 | 237 | 100 (75, 100) | 100 (80, 100) |  |  |  |
|  | 6 | 238 | 79 (67, 100) | 100 (73, 100) | -2.45 | [-6.99, 2.10] | 0.29 |
|  | 12 | 223 | 92 (71, 100) | 100 (79, 100) | -2.83 | [-7.36, 1.69] | 0.22 |
|  | 36 | 237 | 94 (67, 100) | 94 (79, 100) | -3.76 | [-8.54, 1.03] | 0.12 |
|  | 60 | 192 | 100 (73, 100) | 94 (75, 100) | -3.91 | [-8.88, 1.06] | 0.12 |
| Radiation without ADT | 0 | 492 | 100 (85, 100) | 100 (85, 100) |  |  |  |
|  | 6 | 476 | 94 (75, 100) | 100 (79, 100) | -0.6 | [-3.41, 2.22] | 0.68 |
|  | 12 | 462 | 92 (73, 100) | 100 (79, 100) | -0.98 | [-3.70, 1.74] | 0.48 |
|  | 36 | 493 | 92 (67, 100) | 94 (79, 100) | -1.9 | [-4.83, 1.03] | 0.20 |
|  | 60 | 421 | 92 (73, 100) | 100 (79, 100) | -2.06 | [-5.16, 1.04] | 0.19 |
| Active surveillance | 0 | 318 | 100 (75, 100) | 100 (85, 100) |  |  |  |
|  | 6 | 322 | 92 (73, 100) | 100 (85, 100) | -5.96 | [-9.52, -2.39] | <0.01 |
|  | 12 | 310 | 92 (73, 100) | 100 (85, 100) | -6.34 | [-9.77, -2.91] | <0.001 |
|  | 36 | 325 | 79 (59, 100) | 100 (80, 100) | -7.27 | [-10.84, -3.69] | <0.001 |
|  | 60 | 282 | 73 (52, 94) | 100 (79, 100) | -7.42 | [-11.34, -3.50] | <0.001 |
| **Sexual function** | | | | | | | |
| Surgery | 0 | 1231 | 74 (33, 90) | 80 (47, 95) |  |  |  |
|  | 6 | 1204 | 17 (4, 44) | 27 (7, 58) | -2.51 | [-5.68, 0.66] | 0.12 |
|  | 12 | 1210 | 27 (5, 58) | 33 (10, 70) | -2.36 | [-5.33, 0.61] | 0.12 |
|  | 36 | 1256 | 29 (7, 65) | 38 (10, 73) | -2.07 | [-5.23, 1.09] | 0.20 |
|  | 60 | 1098 | 28 (5, 65) | 38 (10, 75) | -2.14 | [-5.60, 1.32] | 0.23 |
| Radiation with ADT | 0 | 223 | 60 (19, 80) | 52 (12, 80) |  |  |  |
|  | 6 | 219 | 5 (0, 32) | 0 (0, 42) | -6.56 | [-12.19, -0.93] | 0.02 |
|  | 12 | 226 | 8 (0, 37) | 17 (0, 58) | -6.41 | [-11.97, -0.86] | 0.02 |
|  | 36 | 224 | 17 (0, 55) | 18 (0, 65) | -6.12 | [-11.84, -0.40] | 0.04 |
|  | 60 | 180 | 27 (0, 55) | 32 (0, 65) | -6.19 | [-12.05, -0.33] | 0.04 |
| Radiation without ADT | 0 | 482 | 63 (26, 85) | 69 (38, 85) |  |  |  |
|  | 6 | 455 | 49 (12, 76) | 58 (27, 81) | -4.51 | [-8.62, -0.41] | 0.03 |
|  | 12 | 466 | 43 (12, 68) | 53 (22, 80) | -4.37 | [-8.32, -0.41] | 0.03 |
|  | 36 | 481 | 43 (7, 70) | 45 (12, 75) | -4.07 | [-8.20, 0.05] | 0.05 |
|  | 60 | 407 | 33 (7, 70) | 38 (10, 73) | -4.15 | [-8.55, 0.26] | 0.07 |
| Active surveillance | 0 | 314 | 75 (42, 85) | 75 (47, 90) |  |  |  |
|  | 6 | 307 | 65 (27, 85) | 75 (43, 92) | -6.76 | [-11.72, -1.80] | 0.01 |
|  | 12 | 298 | 64 (22, 85) | 75 (48, 90) | -6.61 | [-11.47, -1.75] | 0.01 |
|  | 36 | 316 | 38 (10, 78) | 65 (27, 85) | -6.32 | [-11.35, -1.28] | 0.01 |
|  | 60 | 266 | 38 (17, 65) | 63 (27, 85) | -6.39 | [-11.63, -1.15] | 0.02 |
| **Bowel function** | | | | | | | |
| Surgery | 0 | 1262 | 100 (96, 100) | 100 (96, 100) |  |  |  |
|  | 6 | 1232 | 100 (96, 100) | 100 (96, 100) | 1.66 | [0.61, 2.72] | <0.01 |
|  | 12 | 1223 | 100 (96, 100) | 100 (96, 100) | 1.25 | [0.38, 2.13] | 0.01 |
|  | 36 | 1278 | 100 (96, 100) | 100 (96, 100) | 0.64 | [-0.41, 1.70] | 0.23 |
|  | 60 | 1114 | 100 (96, 100) | 100 (96, 100) | 1.31 | [0.15, 2.48] | 0.03 |
| Radiation with ADT | 0 | 239 | 100 (96, 100) | 100 (92, 100) |  |  |  |
|  | 6 | 238 | 100 (83, 100) | 96 (82, 100) | 0.69 | [-2.36, 3.75] | 0.66 |
|  | 12 | 237 | 96 (82, 100) | 92 (79, 100) | 0.29 | [-2.75, 3.32] | 0.85 |
|  | 36 | 242 | 96 (79, 100) | 96 (83, 100) | -0.32 | [-3.44, 2.79] | 0.84 |
|  | 60 | 198 | 96 (83, 100) | 96 (88, 100) | 0.35 | [-2.73, 3.42] | 0.83 |
| Radiation without ADT | 0 | 498 | 100 (92, 100) | 100 (92, 100) |  |  |  |
|  | 6 | 475 | 96 (83, 100) | 96 (88, 100) | 1.27 | [-0.77, 3.30] | 0.22 |
|  | 12 | 481 | 96 (83, 100) | 96 (83, 100) | 0.86 | [-1.10, 2.81] | 0.39 |
|  | 36 | 503 | 100 (83, 100) | 96 (88, 100) | 0.25 | [-1.77, 2.26] | 0.81 |
|  | 60 | 419 | 100 (88, 100) | 96 (88, 100) | 0.92 | [-1.10, 2.93] | 0.37 |
| Active surveillance | 0 | 324 | 100 (92, 100) | 100 (96, 100) |  |  |  |
|  | 6 | 324 | 100 (90, 100) | 100 (96, 100) | -0.41 | [-2.52, 1.70] | 0.70 |
|  | 12 | 318 | 100 (92, 100) | 100 (92, 100) | -0.82 | [-2.88, 1.24] | 0.44 |
|  | 36 | 335 | 96 (89, 100) | 100 (96, 100) | -1.43 | [-3.59, 0.73] | 0.20 |
|  | 60 | 282 | 100 (90, 100) | 100 (96, 100) | -0.76 | [-2.89, 1.38] | 0.49 |
| **Hormone function** | | | | | | | |
| Surgery | 0 | 1241 | 95 (85, 100) | 95 (85, 100) |  |  |  |
|  | 6 | 1212 | 95 (80, 100) | 95 (85, 100) | -2.86 | [-4.32, -1.41] | <0.001 |
|  | 12 | 1211 | 90 (80, 100) | 95 (85, 100) | -2.94 | [-4.25, -1.62] | <0.001 |
|  | 36 | 1263 | 95 (80, 100) | 95 (85, 100) | -3.16 | [-4.60, -1.72] | <0.001 |
|  | 60 | 1105 | 90 (75, 100) | 95 (85, 100) | -3.29 | [-4.84, -1.75] | <0.001 |
| Radiation with ADT | 0 | 233 | 90 (79, 95) | 90 (80, 100) |  |  |  |
|  | 6 | 234 | 75 (60, 85) | 80 (70, 90) | -4.23 | [-7.98, -0.48] | 0.03 |
|  | 12 | 236 | 75 (60, 88) | 85 (70, 95) | -4.3 | [-7.99, -0.62] | 0.02 |
|  | 36 | 235 | 85 (70, 95) | 90 (80, 100) | -4.52 | [-8.24, -0.81] | 0.02 |
|  | 60 | 195 | 85 (70, 95) | 95 (80, 100) | -4.66 | [-8.43, -0.89] | 0.02 |
| Radiation  without ADT | 0 | 484 | 90 (80, 100) | 95 (85, 100) |  |  |  |
|  | 6 | 465 | 95 (80, 100) | 95 (85, 100) | 0.87 | [-0.88, 2.61] | 0.33 |
|  | 12 | 466 | 90 (80, 100) | 95 (85, 100) | 0.79 | [-0.82, 2.41] | 0.34 |
|  | 36 | 496 | 95 (80, 100) | 95 (85, 100) | 0.57 | [-1.09, 2.24] | 0.50 |
|  | 60 | 411 | 95 (81, 100) | 95 (85, 100) | 0.44 | [-1.30, 2.17] | 0.62 |
| Active surveillance | 0 | 321 | 90 (80, 100) | 95 (90, 100) |  |  |  |
|  | 6 | 320 | 95 (85, 100) | 100 (90, 100) | -2.6 | [-4.89, -0.30] | 0.03 |
|  | 12 | 314 | 90 (79, 95) | 95 (90, 100) | -2.67 | [-4.89, -0.46] | 0.02 |
|  | 36 | 331 | 90 (76, 100) | 100 (90, 100) | -2.89 | [-5.21, -0.58] | 0.01 |
|  | 60 | 280 | 90 (75, 95) | 95 (90, 100) | -3.03 | [-5.43, -0.62] | 0.01 |
