# Supplementary material for: Association between body mass index and localized prostate cancer management and disease‐specific quality of life
Source: BJUI Compass. 2022 Nov 2;4(2):223–33. doi: 10.1002/bco2.197 (PMC9931544; doi:10.1002/bco2.197)
Supplement: Supplementary file 4 — Table S4 General health‐related quality of life outcomes: SF‐36 domain scores by obesity status, stratified by management option, adjusted for patient demographic, tumor, and baseline functional characteristics. [file BCO2-4-223-s003.docx]

Supplemental Table 4: General health-related quality of life outcomes: SF-36 domain scores by obesity status, stratified by management option, adjusted for patient demographic, tumor, and baseline functional characteristics.

|  |  |  | Unadjusted median (IQR) | | Obese vs Non-obese | | |
| --- | --- | --- | --- | --- | --- | --- | --- |
| Treatment | Month | N | Obese | Non-obese | Effect | 95% CI | p-value |
| **Physical function** | | | | | | | |
| Surgery | 0 | 1252 | 95 (76, 100) | 100 (90, 100) |  |  |  |
|  | 6 | 1232 | 95 (75, 100) | 95 (90, 100) | -1.41 | [-3.22, 0.39] | 0.13 |
|  | 12 | 1227 | 95 (85, 100) | 100 (90, 100) | -1.06 | [-2.70, 0.58] | 0.21 |
|  | 36 | 1279 | 90 (70, 100) | 95 (85, 100) | -1.27 | [-3.09, 0.55] | 0.17 |
|  | 60 | 1119 | 90 (70, 100) | 95 (85, 100) | -3.52 | [-5.56, -1.47] | <0.001 |
| Radiation | 0 | 729 | 90 (65, 100) | 95 (80, 100) |  |  |  |
|  | 6 | 723 | 85 (65, 95) | 90 (75, 100) | -2.96 | [-5.29, -0.63] | 0.01 |
|  | 12 | 726 | 90 (70, 100) | 95 (80, 100) | -2.6 | [-4.86, -0.34] | 0.02 |
|  | 36 | 754 | 80 (50, 95) | 90 (70, 100) | -2.81 | [-5.31, -0.32] | 0.03 |
|  | 60 | 630 | 80 (50, 90) | 90 (69, 95) | -5.06 | [-7.68, -2.44] | <0.001 |
| Active surveillance | 0 | 313 | 88 (60, 95) | 100 (90, 100) |  |  |  |
|  | 6 | 323 | 85 (65, 100) | 95 (85, 100) | -4.15 | [-7.65, -0.64] | 0.02 |
|  | 12 | 319 | 90 (75, 100) | 100 (90, 100) | -3.79 | [-7.19, -0.39] | 0.03 |
|  | 36 | 333 | 80 (58, 90) | 95 (85, 100) | -4 | [-7.50, -0.50] | 0.03 |
|  | 60 | 285 | 80 (50, 90) | 95 (85, 100) | -6.25 | [-9.97, -2.54] | <0.001 |
| **Emotional well-being** | | | | | | | |
| Surgery | 0 | 1264 | 84 (68, 92) | 84 (68, 92) |  |  |  |
|  | 6 | 1232 | 88 (76, 96) | 88 (76, 92) | 0.28 | [-1.18, 1.74] | 0.71 |
|  | 12 | 1221 | 88 (76, 92) | 88 (73, 92) | -0.05 | [-1.40, 1.29] | 0.94 |
|  | 36 | 1278 | 84 (72, 92) | 84 (76, 92) | -0.44 | [-1.88, 1.00] | 0.55 |
|  | 60 | 1119 | 88 (72, 92) | 88 (76, 92) | 0.34 | [-1.23, 1.90] | 0.67 |
| Radiation | 0 | 740 | 84 (68, 92) | 88 (76, 92) |  |  |  |
|  | 6 | 720 | 84 (72, 92) | 88 (76, 96) | 0.47 | [-1.21, 2.14] | 0.59 |
|  | 12 | 723 | 84 (72, 92) | 88 (76, 92) | 0.13 | [-1.48, 1.74] | 0.87 |
|  | 36 | 750 | 84 (72, 92) | 88 (76, 92) | -0.26 | [-2.02, 1.50] | 0.77 |
|  | 60 | 629 | 84 (68, 92) | 88 (76, 92) | 0.52 | [-1.37, 2.41] | 0.59 |
| Active surveillance | 0 | 320 | 84 (72, 92) | 88 (72, 92) |  |  |  |
|  | 6 | 323 | 88 (76, 92) | 88 (76, 92) | -1.46 | [-3.89, 0.98] | 0.24 |
|  | 12 | 318 | 84 (64, 92) | 88 (80, 92) | -1.79 | [-4.16, 0.58] | 0.14 |
|  | 36 | 332 | 84 (68, 92) | 88 (76, 92) | -2.18 | [-4.63, 0.27] | 0.08 |
|  | 60 | 285 | 80 (68, 92) | 88 (76, 92) | -1.4 | [-3.97, 1.16] | 0.28 |
| **Energy and fatigue** | | | | | | | |
| Surgery | 0 | 1265 | 75 (55, 85) | 80 (65, 90) |  |  |  |
|  | 6 | 1231 | 75 (55, 85) | 80 (65, 85) | -1.91 | [-3.71, -0.11] | 0.04 |
|  | 12 | 1221 | 75 (55, 85) | 80 (65, 85) | -2.19 | [-3.81, -0.57] | 0.01 |
|  | 36 | 1279 | 70 (55, 80) | 75 (60, 85) | -2.66 | [-4.39, -0.92] | 0.00 |
|  | 60 | 1119 | 70 (55, 80) | 75 (60, 85) | -2.29 | [-4.20, -0.38] | 0.02 |
| Radiation | 0 | 741 | 70 (50, 80) | 75 (65, 85) |  |  |  |
|  | 6 | 720 | 65 (50, 80) | 75 (60, 85) | -1.29 | [-3.46, 0.88] | 0.24 |
|  | 12 | 724 | 65 (50, 80) | 75 (60, 85) | -1.57 | [-3.64, 0.50] | 0.14 |
|  | 36 | 750 | 65 (50, 75) | 70 (55, 85) | -2.04 | [-4.27, 0.20] | 0.08 |
|  | 60 | 629 | 65 (50, 80) | 70 (55, 85) | -1.67 | [-3.99, 0.65] | 0.16 |
| Active surveillance | 0 | 320 | 70 (50, 80) | 80 (65, 90) |  |  |  |
|  | 6 | 323 | 70 (50, 80) | 80 (65, 85) | -2.03 | [-4.92, 0.86] | 0.17 |
|  | 12 | 318 | 70 (55, 80) | 80 (65, 85) | -2.31 | [-5.09, 0.46] | 0.10 |
|  | 36 | 332 | 68 (50, 80) | 80 (60, 85) | -2.78 | [-5.62, 0.07] | 0.06 |
|  | 60 | 285 | 65 (55, 80) | 75 (60, 85) | -2.41 | [-5.39, 0.58] | 0.11 |
