# Supplementary material for: Association between body mass index and localized prostate cancer management and disease‐specific quality of life
Source: BJUI Compass. 2022 Nov 2;4(2):223–33. doi: 10.1002/bco2.197 (PMC9931544; doi:10.1002/bco2.197)
Supplement: Supplementary file 5 — Table S5 Internal cohort BMI stability from 3 years to 5 years after baseline [file BCO2-4-223-s004.docx]

Table S5: Internal cohort BMI stability from 3 years to 5 years after baseline

|  | Surgery | Radiation | AS | Combined | P-value |
| --- | --- | --- | --- | --- | --- |
| ∆BMI^a^ |  |  |  |  | 0.09 |
| >=5% increase | 153 (14%) | 78 (13%) | 28 (10%) | 259 (13%) |  |
| <5% change | 811 (73%) | 441 (71%) | 207 (73%) | 1459 (73%) |  |
| >=5% decrease | 142 (13%) | 103 (16%) | 47 (17%) | 292 (14%) |  |

^a^The change is BMI (∆BMI) is defined as the difference between BMI at 5 years and 3 years after baseline, then categorized into three groups: significant increase, stable, or significant decrease, where 5% change represents a clinically significant difference. Of 2378 study participants, 2010 had BMI data at 5 years.
