# Supplementary material for: Association between body mass index and localized prostate cancer management and disease‐specific quality of life
Source: BJUI Compass. 2022 Nov 2;4(2):223–33. doi: 10.1002/bco2.197 (PMC9931544; doi:10.1002/bco2.197)
Supplement: Supplementary file 6 — Table S6 Multinomial multivariable logistic regression model for the outcome of management option including all covariates. [file BCO2-4-223-s005.docx]

Supplemental Table 6

Multinomial multivariable logistic regression model for the outcome of management option including all covariates.

|  | RP vs RT | | | AS vs RT | | |
| --- | --- | --- | --- | --- | --- | --- |
|  | Relative Risk Ratio | 95% Confidence Interval | p-value | Relative Risk Ratio | 95% Confidence Interval | p-value |
| BMI at 3 year restricted-cubic-spline | 0.93 | (0.87, 0.99) | **0.021** | 0.98 | (0.90, 1.06) | 0.639 |
| Age at diagnosis - (59, 69) | 0.30 | (0.24, 0.37) | **<.001** | 0.92 | (0.71, 1.18) | 0.502 |
| Race: White reference |  |  |  |  |  |  |
| Black | 0.59 | (0.42, 0.83) | **0.003** | 1.06 | (0.66, 1.69) | 0.818 |
| Hispanic | 0.88 | (0.52, 1.49) | 0.633 | 0.92 | (0.44, 1.92) | 0.829 |
| Asian | 1.16 | (0.59, 2.27) | 0.668 | 0.73 | (0.28, 1.87) | 0.51 |
| Other | 0.93 | (0.38, 2.29) | 0.874 | 0.92 | (0.27, 3.09) | 0.891 |
| Education: Less than high school reference |  |  |  |  |  |  |
| High school graduate | 1.03 | (0.65, 1.65) | 0.894 | 1.21 | (0.61, 2.39) | 0.579 |
| Some college | 0.89 | (0.55, 1.45) | 0.643 | 1.17 | (0.59, 2.35) | 0.653 |
| College graduate | 0.86 | (0.52, 1.44) | 0.576 | 1.25 | (0.61, 2.56) | 0.534 |
| Graduate/professional | 1.00 | (0.59, 1.68) | 0.997 | 1.52 | (0.74, 3.12) | 0.25 |
| Marital status - Married vs Not Married | 1.78 | (1.33, 2.37) | **<.001** | 1.32 | (0.89, 1.98) | 0.169 |
| Comorbidity: TIBI 0-2 reference |  |  |  |  |  |  |
| 3-4 | 0.99 | (0.75, 1.30) | 0.942 | 0.87 | (0.60, 1.25) | 0.441 |
| 5 or more | 0.75 | (0.54, 1.03) | 0.072 | 0.98 | (0.63, 1.51) | 0.922 |
| Income: Less than $30,000 reference |  |  |  |  |  |  |
| $30,001 - $50,000 | 1.15 | (0.78, 1.68) | 0.482 | 1.1 | (0.66, 1.85) | 0.709 |
| $50,001 - $100,000 | 1.23 | (0.83, 1.81) | 0.3 | 1.13 | (0.65, 1.95) | 0.66 |
| >$100,000 | 1.16 | (0.75, 1.80) | 0.5 | 1.2 | (0.66, 2.18) | 0.547 |
| Health insurance: Medicare reference |  |  |  |  |  |  |
| Private/HMO | 1.23 | (0.92, 1.63) | 0.161 | 1.15 | (0.78, 1.68) | 0.483 |
| VA/military/Medicaid/other/none | 0.90 | (0.52, 1.53) | 0.685 | 1.41 | (0.67, 2.97) | 0.365 |
| Employment: Full time reference |  |  |  |  |  |  |
| Part time | 1.01 | (0.67, 1.54) | 0.955 | 1.53 | (0.87, 2.66) | 0.137 |
| Retired | 0.92 | (0.68, 1.23) | 0.56 | 1.09 | (0.73, 1.65) | 0.666 |
| Unemployed | 0.78 | (0.45, 1.35) | 0.377 | 0.95 | (0.42, 2.12) | 0.894 |
| PSA at diagnosis - (4, 7) | 0.94 | (0.89, 1.00) | 0.061 | 0.92 | (0.83, 1.02) | 0.113 |
| Clinical tumor stage - T2 vs T1 | 1.24 | (0.98, 1.59) | 0.077 | 0.82 | (0.56, 1.19) | 0.291 |
| Biopsy Gleason score: 6 or less reference |  |  |  |  |  |  |
| 3 + 4 = 7 | 0.94 | (0.74, 1.20) | 0.638 | 0.14 | (0.09, 0.21) | **<.001** |
| 4 + 3 = 7 | 1.25 | (0.89, 1.77) | 0.202 | 0.06 | (0.02, 0.14) | **<.001** |
| 8 to 10 | 0.91 | (0.64, 1.30) | 0.611 | 0.04 | (0.01, 0.11) | **<.001** |
| Site: Utah reference |  |  |  |  |  |  |
| Atlanta | 0.47 | (0.30, 0.72) | **<.001** | 0.25 | (0.14, 0.45) | **<.001** |
| LA | 1.78 | (1.15, 2.75) | **0.009** | 1.06 | (0.63, 1.81) | 0.817 |
| Louisiana | 1.02 | (0.68, 1.54) | 0.915 | 0.54 | (0.33, 0.90) | 0.018 |
| NJ | 0.71 | (0.46, 1.09) | 0.117 | 0.2 | (0.11, 0.35) | **<.001** |
| CaPSURE | 1.86 | (1.02, 3.41) | **0.044** | 1.24 | (0.58, 2.64) | 0.571 |
| SF36 physical functioning at baseline - (85, 100) | 0.98 | (0.89, 1.08) | 0.721 | 0.87 | (0.76, 0.99) | **0.038** |
| SF36 emotional well-being at baseline - (72, 92) | 0.75 | (0.60, 0.93) | **0.009** | 0.9 | (0.67, 1.20) | 0.464 |
| SF36 energy and fatigue at baseline - (60, 85) | 1.21 | (0.98, 1.50) | 0.083 | 1.1 | (0.82, 1.47) | 0.533 |
| Social support at baseline - (70, 100) | 1.05 | (0.93, 1.19) | 0.435 | 1.06 | (0.90, 1.26) | 0.475 |
| Center for Epi. Studies Depression Scale at baseline - (4, 30) | 1.21 | (0.92, 1.58) | 0.177 | 0.98 | (0.69, 1.40) | 0.91 |
| Participatory decision-making scale at baseline - (71, 93) | 1.12 | (1.00, 1.26) | **0.045** | 0.92 | (0.80, 1.08) | 0.31 |
| EPIC-26 urinary irritative domain score at baseline - (75, 100) | 0.85 | (0.70, 1.03) | 0.09 | 1.03 | (0.79, 1.34) | 0.814 |
| EPIC-26 urinary incontinence domain score at baseline - (85, 100) | 0.79 | (0.71, 0.87) | **<.001** | 0.92 | (0.80, 1.06) | 0.266 |
| EPIC-26 bowel function score at baseline - (95, 100) | 0.99 | (0.94, 1.04) | 0.719 | 0.96 | (0.89, 1.03) | 0.243 |
| EPIC-26 sexual function score at baseline - (38, 90) | 1.06 | (0.87, 1.28) | 0.592 | 1.55 | (1.18, 2.05) | **0.002** |
| EPIC-26 hormonal domain score at baseline - (85, 100) | 1.48 | (1.24, 1.78) | **<.001** | 1.15 | (0.91, 1.46) | 0.243 |
