# Supplementary figures and images for: Association between body mass index and localized prostate cancer management and disease‐specific quality of life
Source: BJUI Compass. 2022 Nov 2;4(2):223–33. doi: 10.1002/bco2.197 (PMC9931544; doi:10.1002/bco2.197)

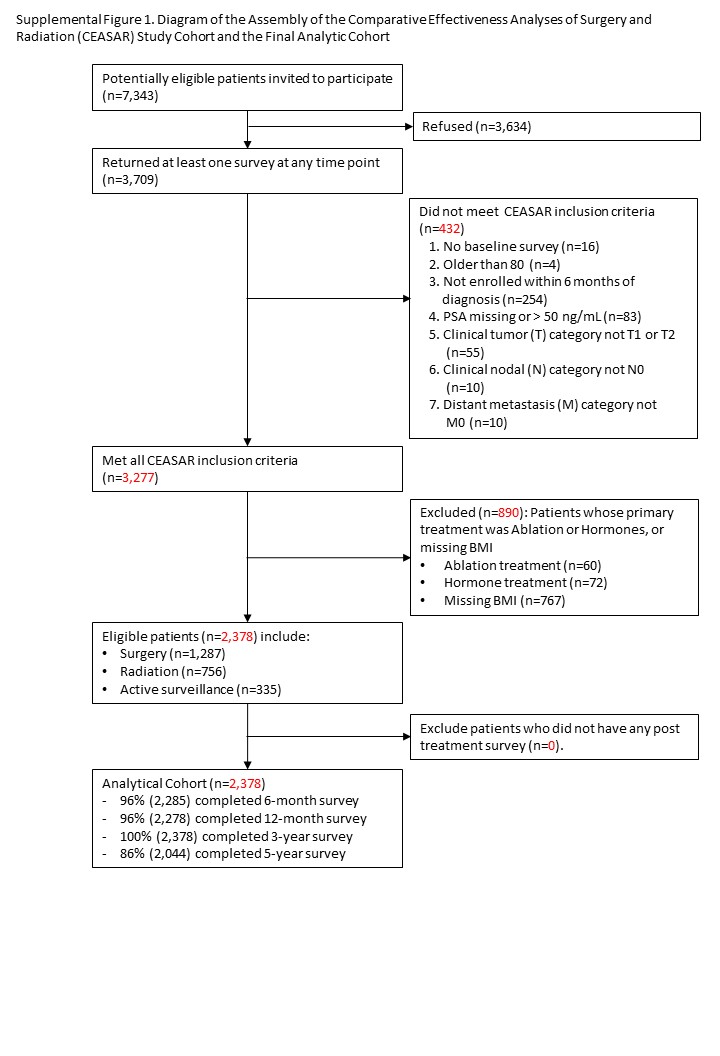

Supplement: Supplementary file 7 — Figure S1: Diagram of the assembly of the Comparative Effectiveness Analysis of Surgery and Radiation (CEASAR) study cohort and the final analytic cohort [file BCO2-4-223-s001.jpg]

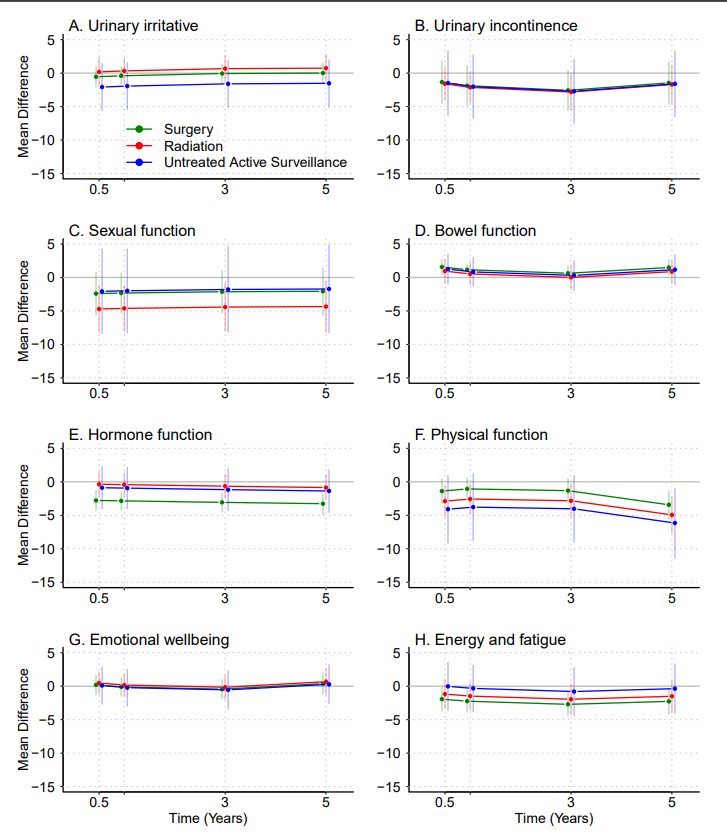

Supplement: Supplementary file 8 — Figure S2: Adjusted EPIC‐26 domain score mean‐differences between obese and non‐obese men, stratified by management option, over time in an as‐treated analysis (i.e. excluding men initially on AS who underwent later definitive treatment) [file BCO2-4-223-s002.png]
